# Deep learning parametric response mapping from inspiratory chest CT scans: a new approach for small airway disease screening

**DOI:** 10.1186/s12931-023-02611-2

**Published:** 2023-11-28

**Authors:** Bin Chen, Ziyi Liu, Jinjuan Lu, Zhihao Li, Kaiming Kuang, Jiancheng Yang, Zengmao Wang, Yingli Sun, Bo Du, Lin Qi, Ming Li

**Affiliations:** 1 Department of Radiology, Huadong Hospital Affiliated to Fudan University, 221, Yanan West Road, Jingan Temple Street, Jingan District, Shanghai, China; 2 Zhang Guozhen Small Pulmonary Nodules Diagnosis and Treatment Center, Shanghai, China; 3 School of Computer Science, Wuhan University, LuoJiaShan, WuChang District, Wuhan, Hubei China; 4 Artificial Intelligence Institute of Wuhan University, Wuhan, Hubei China; 5 Hubei Key Laboratory of Multimedia and Network Communication Engineering, Wuhan, Hubei China; 6 Department of Radiology, Shanghai Geriatric Medical Center, Shanghai, China; 7 Dianei Technology, Shanghai, China; 8 University of California San Diego, La Jolla, USA; 9 Computer Vision Laboratory, Swiss Federal Institute of Technology Lausanne (EPFL), Lausanne, Switzerland

**Keywords:** Computed tomography, Deep learning, Parametric response mapping, Small airways

## Abstract

**Objectives:**

Parametric response mapping (PRM) enables the evaluation of small airway disease (SAD) at the voxel level, but requires both inspiratory and expiratory chest CT scans. We hypothesize that deep learning PRM from inspiratory chest CT scans can effectively evaluate SAD in individuals with normal spirometry.

**Methods:**

We included 537 participants with normal spirometry, a history of smoking or secondhand smoke exposure, and divided them into training, tuning, and test sets. A cascaded generative adversarial network generated expiratory CT from inspiratory CT, followed by a UNet-like network predicting PRM using real inspiratory CT and generated expiratory CT. The performance of the prediction is evaluated using SSIM, RMSE and dice coefficients. Pearson correlation evaluated the correlation between predicted and ground truth PRM. ROC curves evaluated predicted PRM^fSAD^ (the volume percentage of functional small airway disease, fSAD) performance in stratifying SAD.

**Results:**

Our method can generate expiratory CT of good quality (SSIM 0.86, RMSE 80.13 HU). The predicted PRM dice coefficients for normal lung, emphysema, and fSAD regions are 0.85, 0.63, and 0.51, respectively. The volume percentages of emphysema and fSAD showed good correlation between predicted and ground truth PRM (|r| were 0.97 and 0.64, respectively, *p* < 0.05). Predicted PRM^fSAD^ showed good SAD stratification performance with ground truth PRM^fSAD^ at thresholds of 15%, 20% and 25% (AUCs were 0.84, 0.78, and 0.84, respectively, *p* < 0.001).

**Conclusion:**

Our deep learning method generates high-quality PRM using inspiratory chest CT and effectively stratifies SAD in individuals with normal spirometry.

**Supplementary Information:**

The online version contains supplementary material available at 10.1186/s12931-023-02611-2.

Chronic respiratory diseases are closely related to public health issues such as tobacco smoke, secondhand smoke, and environmental pollution, and remain a leading cause of disability and death worldwide [1–3]. Small airway disease (SAD) is one of its pathological bases. Before the onset of chronic obstructive pulmonary disease (COPD), emphysema or clinical symptoms, histology and micro-CT have shown extensive narrowing and destruction of small airways (< 2 mm) [4–6]. By reducing exposure to risk factors, disease progression can be effectively prevented. Under normal conditions, small airways account for less than 25% of total airflow resistance, and SAD can accumulate unnoticed. Spirometry is not sensitive enough to detect SAD, and it takes the loss of about one-third of small airways to affect FEV1/FVC. Although high-resolution computed tomography (HRCT) can show emphysema and large airway abnormalities, its resolution is limited and cannot directly observe small airways unless exudative inflammatory SAD is present [7, 8].

SAD can result in air trapping, observable on expiratory CT, with the degree of air trapping demonstrating a strong correlation with functional airway obstruction [9, 10]. Galbán et al. [11] proposed a method known as parametric response mapping (PRM), which is based on co-registered paired inspiratory-expiratory HRCT series. This method is capable of quantifying the proportion of air trapping caused by emphysema and functional small airway disease (fSAD), and generating a visual map. PRM has been utilized to evaluate COPD, asthma, and SAD, assess disease progression and drug response [12–15]. However, expiratory CT is not used for routine clinical examinations, and two chest CT scans increase examination time, radiation dose, and cost, limiting PRM's clinical utility in large-scale screening.

The importance of dual-phase CT for diseases involving the airway, particularly SAD, is self-evident, and it is likely to become increasingly prevalent as a routine examination for such diseases in the future. However, addressing the need for two CT scans has become a significant research issue. Deep learning-based methods have demonstrated exceptional performance in challenging tasks such as disease classification [16, 17], image segmentation [18] and image registration [19], providing the necessary conditions for resolving this issue. Given the widespread use of conventional inspiratory CT scanning protocols in scenarios such as physical examinations and cancer screening, we hypothesize that deep learning-based methods can directly generate PRM from single inspiratory HRCT scans and effectively stratify SAD in populations with normal spirometry exposed to smoking or secondhand smoke. To our knowledge, no existing research utilizes only inspiratory CT scans for voxel-level diagnosis of SAD.

## Materials and methods

### Study participants

**Fig. 1:**
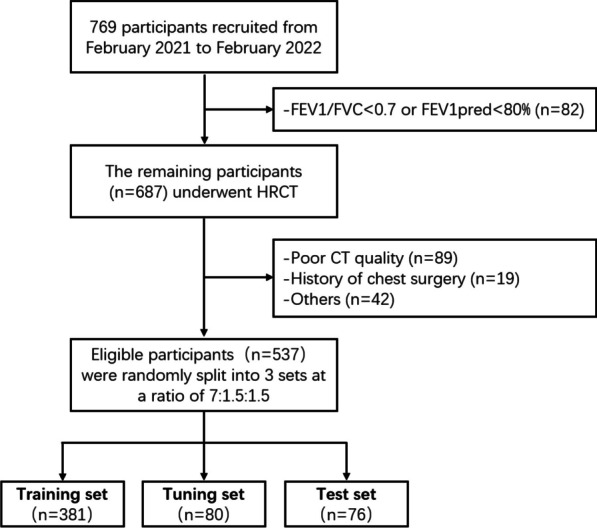
Flowchart of participant inclusion and exclusion. Individuals with FEV1/FVC < 0.7 or FEV1pred < 80% were excluded from the study (n = 82). Those who showed poor cooperation during CT scans, insufficient expiration and inspiration, or motion artifacts on the image were not considered for the study (n = 89). Furthermore, individuals with a history of thoracic surgery (n = 19) and other conditions (n = 42) such as a huge thoracic mass, severe interstitial lung disease, etc., as observed on CT, were also not included in our study. Eligible participants were randomly divided into training, tuning, and test set using a random number generator. We used approximately 70% of the data for model training, 15% for model tuning, and the remaining 15% for performance testing. FEV1: forced expiratory volume in the first second; FVC: forced vital capacity

We prospectively recruited 769 participants (February 2021 to February 2022) who underwent routine health check-ups at our hospital and had at least 5 years of smoking or secondhand smoke exposure history. Participants underwent pulmonary function tests (PFTs) according to guidelines from the American Thoracic Society and the European Respiratory Society [20], and had inspiratory and expiratory HRCT scans within 2 weeks. The study was approved by the institutional ethics committee of our hospital (No. 2021K018), and informed consent was obtained from all participants. The participant recruitment flowchart is shown in Fig. 1.

### HRCT imaging and visual evaluation

Prior to CT scanning, all participants were informed of the purpose and procedure of the examination and underwent multiple respiratory trainings. A supine position was assumed and volumetric thin-slice chest scans were performed on participants in a fully inhaling and exhaling state using a dual-source CT system (Somatom Definition Flash, Siemens Healthcare, Forchheim, Germany). The scan parameters were recommended by the Fleischner Society [21]: Pitch: 1.0; Acquisition collimation: ≤ 1 mm; Kilovolt peak: 120; Effective milliampere second: 40–200; Reconstruction section thickness: 0.625–1 mm. Data from the thoracic inlet to the lung base were reconstructed using the B30f kernel.

Referring to the Fleischner Society statement [21], we visually evaluated participants' lung HRCT for emphysema, inflammatory SAD, bronchial wall thickening, and tracheobronchial dilation. Centrilobular emphysema (CLE) was divided into trace, mild, moderate, confluent, and advanced destructive emphysema (ADE), with scores of 1 to 5 respectively. Inflammatory SAD was defined as ill-defined ground-glass centrilobular nodules [7, 8, 21]. We semi-quantitatively evaluated the extent of inflammatory SAD and bronchial dilation using three degrees of severity (see Additional file 1). CT images were independently evaluated by two radiologists blinded to participants' clinical information. Any discrepancies were resolved through discussion.

## PRM generation model

### Overview

**Fig. 2:**
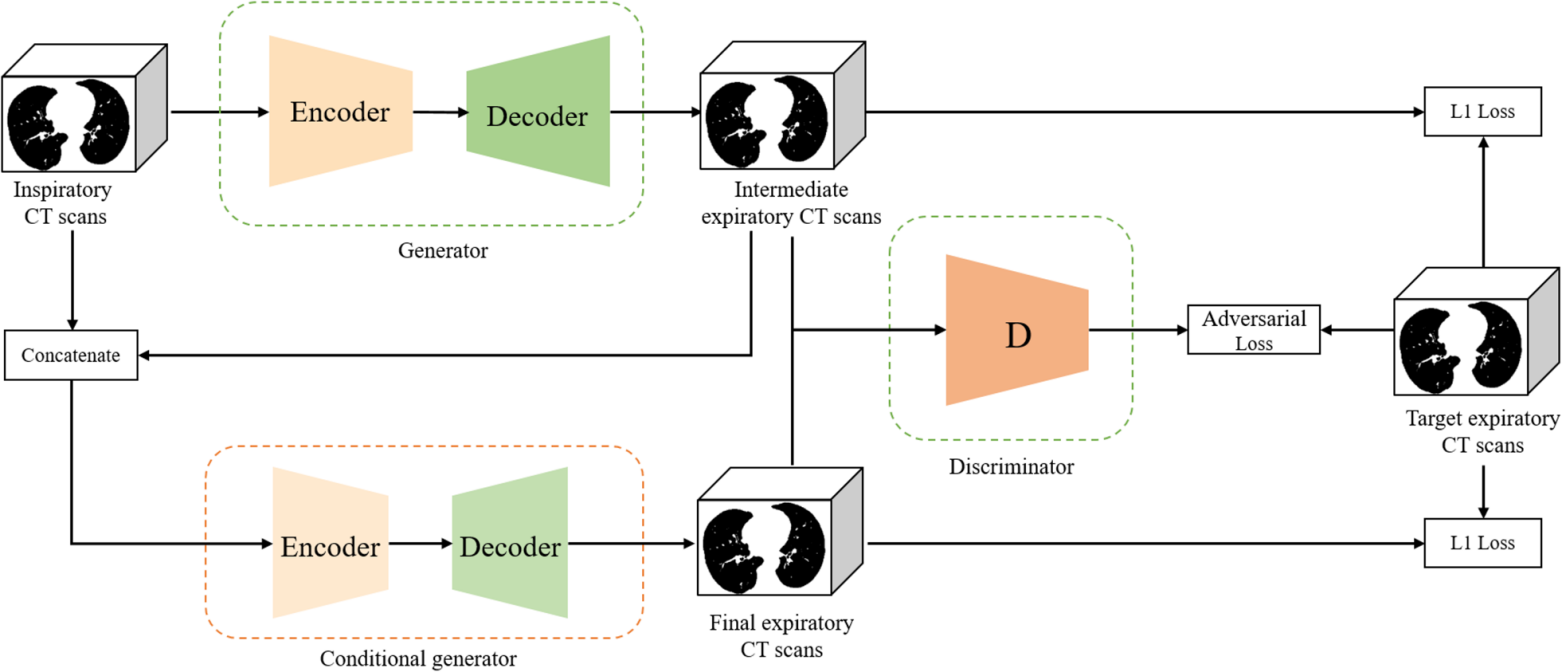
The structure of the expiratory generator. The expiratory generator consists of two parts: a generator that produces coarse expiratory CT scans, and a conditional generator that refines them. Both subnetworks have UNet-like structures and share a discriminator that calculates adversarial loss by comparing the generated CT scans with real expiratory CT scans. Additionally, L1 loss is used to supervise each generator

**Fig. 3:**
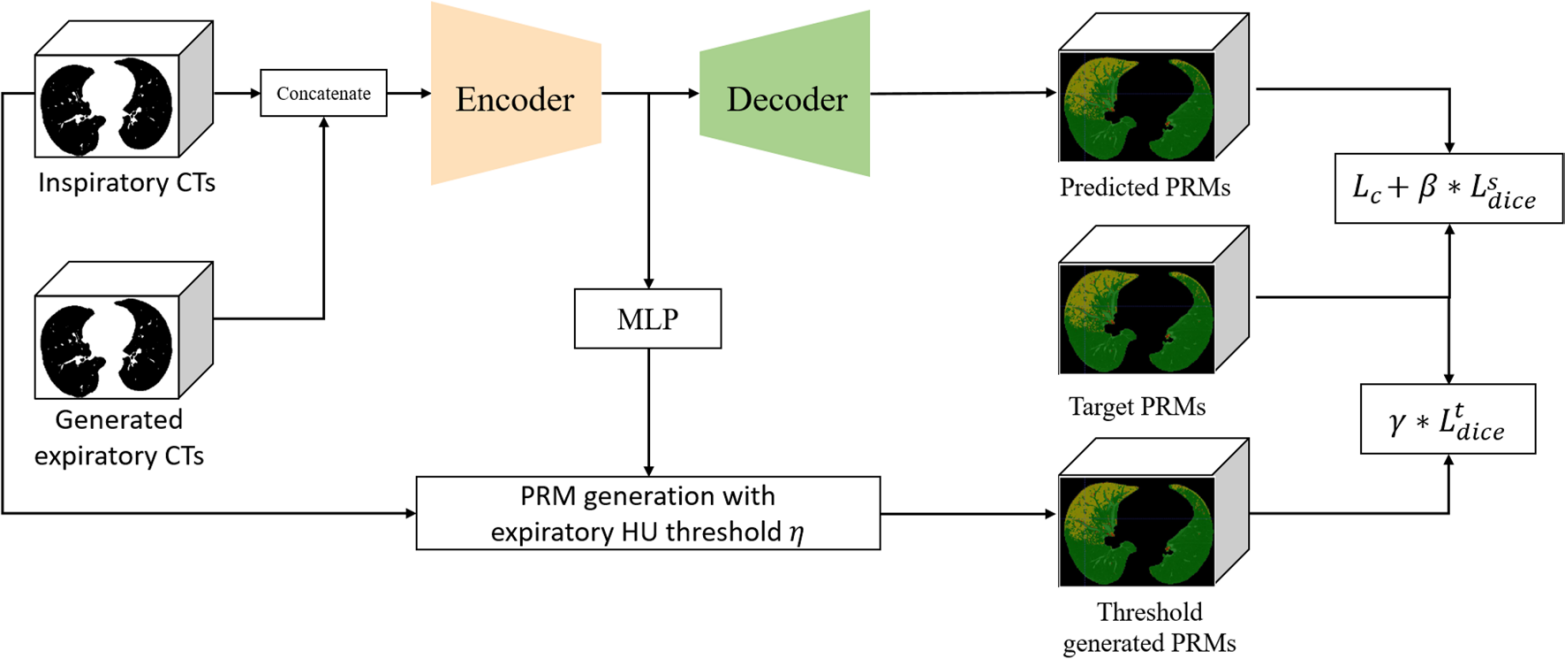
The structure of the PRM generator. The expiratory generator has a UNet-like structure and includes an auxiliary multilayer perceptron (MLP) layer that produces a learnable HU threshold. The PRMs generated using this threshold are then used to calculate the consistency loss

Our model’s function is to generate PRM predictions from inspiratory CT scans. It consists of two networks: an expiratory generator and a PRM generator (details in Additional file 1). The expiratory generator learns the mapping function from the inspiratory domain to the expiratory domain using generative adversarial networks (GANs) to produce expiratory CT scans that are registered with real ones. To reduce local HU errors and global structural errors between reconstructed and real expiratory CT scans, we use an encoder-decoder network [22] to learn local HU residual errors and predict the segmentation of different lesion areas (Fig. 2). Finally, the PRM predicted by the encoder-decoder network is combined with the PRM generated by the predicted expiratory threshold to produce the final PRM prediction (Fig. 3).

### PRM label generation

To obtain the ground truth PRM, we use the same method proposed in a previous study [11]. Specifically, PRM voxels are classified as normal parenchyma, fSAD, or emphysema. First, we use a well-trained U-net model [23] to segment the lung area. Then, using the Free Form Deformation (FFD) non-rigid algorithm, specifically the registration function packaged in the Simple Elastix library in Python, expiratory CT scans were spatially registered to align with inspiratory CT scans on each voxel. The presence of fSAD is determined by lung voxels with inspiratory attenuation > − 950 HU and expiratory attenuation < − 856 HU. Emphysema lesions are identified by lung voxels with inspiratory attenuation < − 950 HU and expiratory attenuation < − 856 HU. All other regions in the lung are considered normal parenchyma. To evaluate the accuracy of the ground truth PRM in this study, we also compare our results with those obtained using commercial software (Aview, Coreline Soft, Seoul, Korea).

### Evaluation metrics

For the generated expiratory CT scans, the structural and local error are both taken into consideration. In this paper, structural similarity index (SSIM) [24] is used to measure CT scans similarity from brightness, contrast and structure:

$$SSIM=\frac{(2{\mu }_{x}{\mu }_{z}+{C}_{1})(2{\sigma }_{xz}+{C}_{2})}{({\mu }_{x}^{2}{+\mu }_{z}^{2}+{C}_{1})({\sigma }_{x}^{2}{+\sigma }_{z}^{2}+{C}_{2})}$$

where $x$ and $z$ are the inspiratory and generated expiratory CT scans, ${\mu }_{x}$ and ${\mu }_{z}$ is the mean voxel value of $x$ and $z$, ${\sigma }_{x}^{2}$ and ${\sigma }_{z}^{2}$ are the variance of $x$ and $z$, ${\sigma }_{xz}$ is the covariance of $x$ and $z$, ${C}_{1}$ and ${C}_{2}$ are the basic constants to keep the denominator from being 0.

We choose Root Mean Squared Error (RMSE) to measure the local voxel value error. RMSE for real and generated expiratory CT scans is defined as follow:

$$RMSE\left(X,Y\right)=\sqrt{\frac{1}{N\times \left|V\right|}{\Sigma }_{i}^{N}{\Sigma }_{\mathrm{v}\in \mathrm{V}}{\left({x}_{iv}-{z}_{iv}\right)}^{2}}$$

where N is the batch size of CT scans, $V$ is the voxel set for each CT scan.

Dice coefficient is chosen to evaluate the performance of our segmentation network. For label $c$, the dice coefficient is the positive area of overlap divided by the total number of pixels in the target and predicted PRM:

$${Dice}_{c}=\frac{\left|{Y}_{c}\bigcap {\widehat{Y}}_{c}\right|}{\left|{Y}_{c}\right|+\left|{\widehat{Y}}_{c}\right|}$$

where $\left|{Y}_{c}\bigcap {\widehat{Y}}_{c}\right|$ is the number of pixels in the overlapping area of label $c$, $\left|{Y}_{c}\right|$ and $\left|{\widehat{Y}}_{c}\right|$ are the number of label $c$ pixels in the target and predicted PRM.

### Statistical analysis

We conducted a Pearson correlation analysis of the PRM parameters and their correlation with visual evaluation. PRM^fSAD^ is generally considered to have significant SAD above 15–25% [15, 25–27]. We used R software (Windows version 4.3.0) to binarize GT PRM^fSAD^ (with values ranging from 10 to 30%) and calculated the AUC value between binarized GT PRM^fSAD^ and Pred PRM^fSAD^ (see Additional file 1: Table S1). The classification performance of Pred PRM^fSAD^ was evaluated via receiver operating characteristic (ROC) curves. According to the AUC value and our data distribution, we chose 15% as the GT PRM^fSAD^ threshold for SAD stratification, at which point the Pred PRM^fSAD^ was also stratified using this cut-off value, and the characteristics of both groups were compared. Categorical variables were expressed as frequency (percentage) and subjected to analysis using either chi-square test or Fisher’s exact test. Continuous variables were expressed as mean ± standard deviation (SD) and analyzed using one-way analysis of variance (ANOVA). In cases where variances were not equal, Brown-Forsythe anova test was employed. Statistical analyses were conducted using SPSS (version 23.0 for Windows; SPSS, Chicago, IL, USA), with *p* < 0.05 considered statistically significant.

## Results

### Participant characteristics

Of the 769 participants recruited, 537 (263 females and 274 males) were included in the study after exclusion criteria (Fig. 1). These participants, with a history of smoking or secondhand smoke exposure for over 5 years, had normal spirometry and an average age of 62.2 (range 41–85 years) (Additional file 1: Table S2). They were randomly divided into training, tuning, and test sets.

The CT images of the 76 test set participants were visually assessed by radiologists. Surprisingly, even with normal spirometry, these tobacco smoke-exposed participants had a considerable proportion of CLE (63.2%) and inflammatory SAD (38.1%) (Table 1). CLE was mostly seen in trace and mild amounts; moderate and confluent were rare. Inflammatory SAD was most common in single lung segment involvement, followed by multiple lung segments, and extensive lung segments were rare. Mild and substantial paraseptal emphysema were also common (Table 1).

**Table 1:** Visual assessment of CT for test set participants

	Total	GT PRM^fSAD^ > 15%	GT PRM^fSAD^ ≤ 15%
No. of subjects	76	46	30
CLE			
Total	68 (63.2)	33 (71.7)	15 (50)
Trace	26 (34.2)	16 (34.8)	10 (33.4)
Mild	16(21.1)	12 (26)	4 (13.3)
Moderate	5 (6.6)	5 (10.9)	0 (0)
Confluent	1 (1.3)	0 (0)	1 (3.3)
ADE	0 (0)	0 (0)	0 (0)
Panlobular emphysema			
No	73 (96.1)	44 (95.7)	29 (96.7)
Yes	3 (3.9)	2 (4.3)	1 (3.3)
Paraseptal emphysema			
Total	21 (27.7)	16 (34.8)	5 (16.7)
Mild	11 (14.5)	9 (19.6)	2 (6.7)
Substantial	10 (13.2)	7 (15.2)	3 (10)
Bronchial wall thickening			
No	72 (94.7)	44 (95.7)	28 (93.3)
Yes	4 (5.3)	2 (4.3)	2 (6.7)
Bronchial dilation			
Total	24 (31.6)	17 (37)	7 (23.3)
Single Lung Segment	19 (25)	13 (28.3)	6 (20)
Multiple Lung Segments	5 (6.6)	4 (8.7)	1 (3.3)
Extensive Lung Segments	0 (0)	0 (0)	0 (0)
Inflammatory SAD			
Total	29 (38.1)	25 (54.3)	4 (13.4)
Single Lung Segment	19 (25)	17 (36.9)	2 (6.7)
Multiple Lung Segments	9 (11.8)	7 (15.2)	2 (6.7)
Extensive Lung Segments	1 (1.3)	1 (2.2)	0 (0)

Data are presented as numbers, with percentages in parentheses

PRM: parametric response mapping; GT: ground truth; fSAD: functional small airway disease; SAD: small airway disease; CLE: centrilobular emphysema; ADE: advanced destructive emphysema

### Evaluation of PRM

We conducted an ablation study to verify the effectiveness of our proposed structures (Table 2). For expiratory CT generation, the cascade GAN structure (vanilla GAN + conditional generator) achieved better SSIM (0.86) and RMSE (80.13 HU) than a vanilla GAN, indicating that the generated expiratory CT scans have strong structural similarity to real images, which improved the performance of the generated PRM.

**Table 2:** Evaluation metrics of predicted images obtained through different methods (n = 76)

Methods	Under different methods				Predicted expiratory CT		Predicted PRM		
	GAN	Conditional generator	PRM generator	Learnable threshold	SSIM	RMSE (HU)	Dice (PRM^Normal^)	Dice (PRM^fSAD^)	Dice (PRM^Emph^)
SegmentNet with inspiratory			√		…	…	0.84	0.27	0.23
Add GAN	√		√		0.80	88.10	0.85	0.42	0.45
Add Conditional generator	√	√	√		0.86	80.13	0.86	0.48	0.54
Threshod PRM results	√	√			0.86	80.13	0.86	0.45	0.50
Add threshod segmentNet	√	√	√	√	0.86	80.13	0.85	0.51	0.63

GAN: generative adversarial network; PRM: parametric response mapping; fSAD: functional small airway disease; PRM^Normal^: the volume percentage of normal area in PRM; PRM^fSAD^: the volume percentage of fSAD in PRM; PRM^Emph^: the volume percentage of emphysema in PRM; SSIM: structural similarity index; RMSE: root mean squared error

**Fig. 4:**
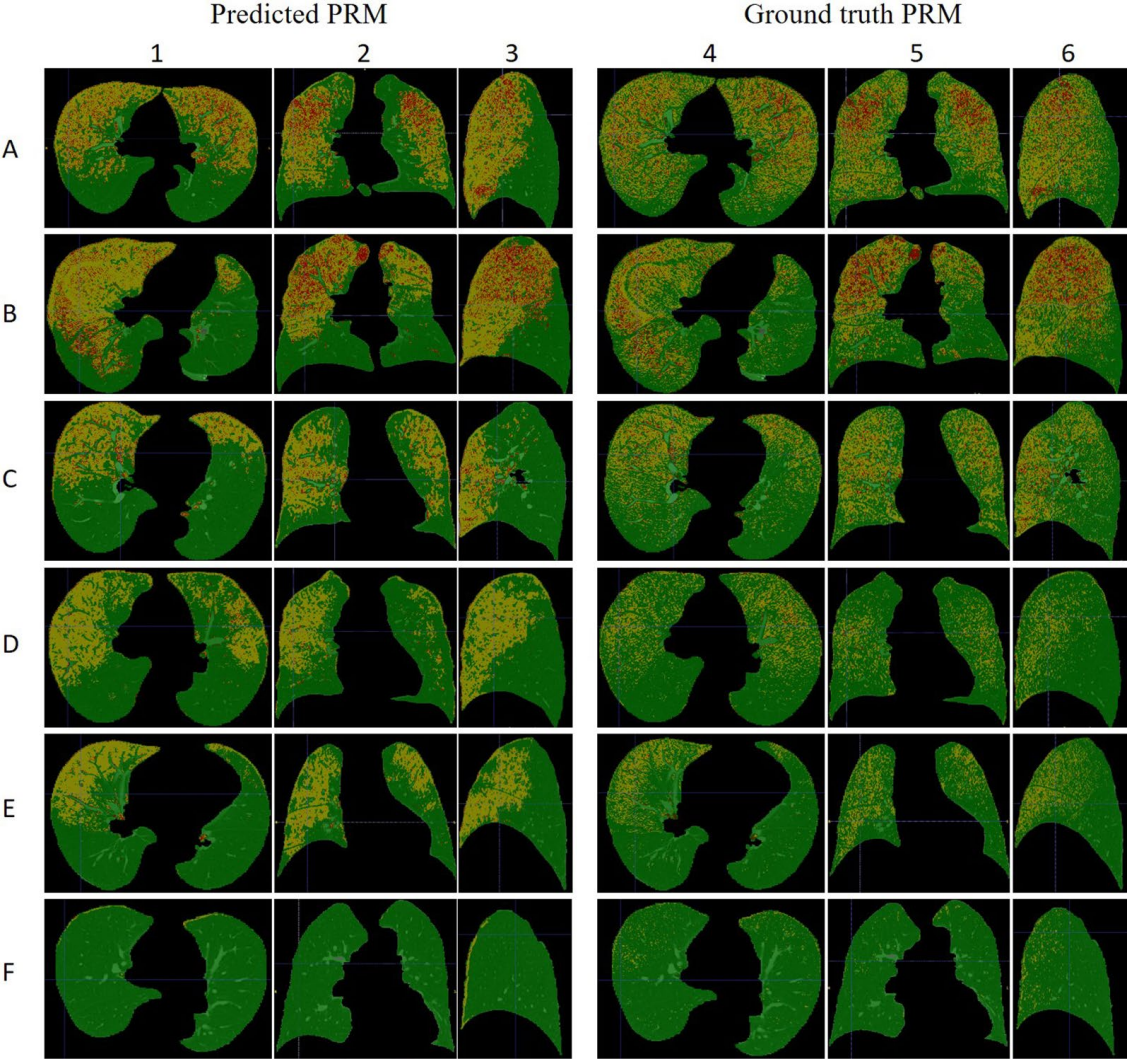
Representative predicted and ground truth PRMs of 6 research participants (**A–F**) in the test set. The left 3 columns (**1–3**) are predicted PRMs based on single inspiratory chest CT scan using deep learning, and the right 3 columns (**4–6**) are ground truth PRMs from real inspiratory and expiratory CT scans. In the PRM, red represents emphysema, yellow represents fSAD, and green represents normal areas. Participants **A** and **B** have moderate CLE on CT with bronchial dilation and inflammatory SAD. Both predicted and ground truth PRMs show a large range of emphysema and fSAD areas (GT PRM^Emph^, Pred PRM^Emph^, GT PRM^fSAD^, Pred PRM^fSAD^ for participant **A** were 7.1%, 8%, 35%, 35.8%, respectively, and for participant **B** were 8%, 8.9%, 29.5%, 32.9%, respectively). Participant **C** has only focal CLE on CT with slight bronchiectasis and no inflammatory SAD, but both predicted and ground truth PRMs show a not small fSAD area (GT PRM^fSAD^, Pred PRM^fSAD^ being 27.9%, 35.9%, respectively). Participants **D–F** have no abnormalities on CT. Participants **D** and **E** still have fSAD areas in PRM, but their lesion volume percentages are not high (GT PRM^fSAD^, Pred PRM^fSAD^ for participant **D** were 11.3%, 17.1%, respectively; GT PRM^fSAD^, Pred PRM^fSAD^ for participant **E** were 12%, 16.7%, respectively). Participant **F** has a perfect lung both visually and quantitatively. Overall, the similarity between the emphysema regions in the predicted and ground truth PRMs is high, but the predicted PRM has relatively less fSAD in dependent lung areas and more fSAD in non-dependent lung areas

The PRM generated by a PRM generator consisting only of SegmentNet with inspiratory had a low dice coefficient, indicating that the PRM generator cannot function well without expiratory generators. Finally, we used a PRM generator with a learnable threshold, further increasing the dice coefficient of both emphysema and fSAD (0.63 and 0.51 respectively) in the generated PRM, allowing for clear localization of different lesion types in the predicted PRM (Fig. 4).

**Fig. 5:**
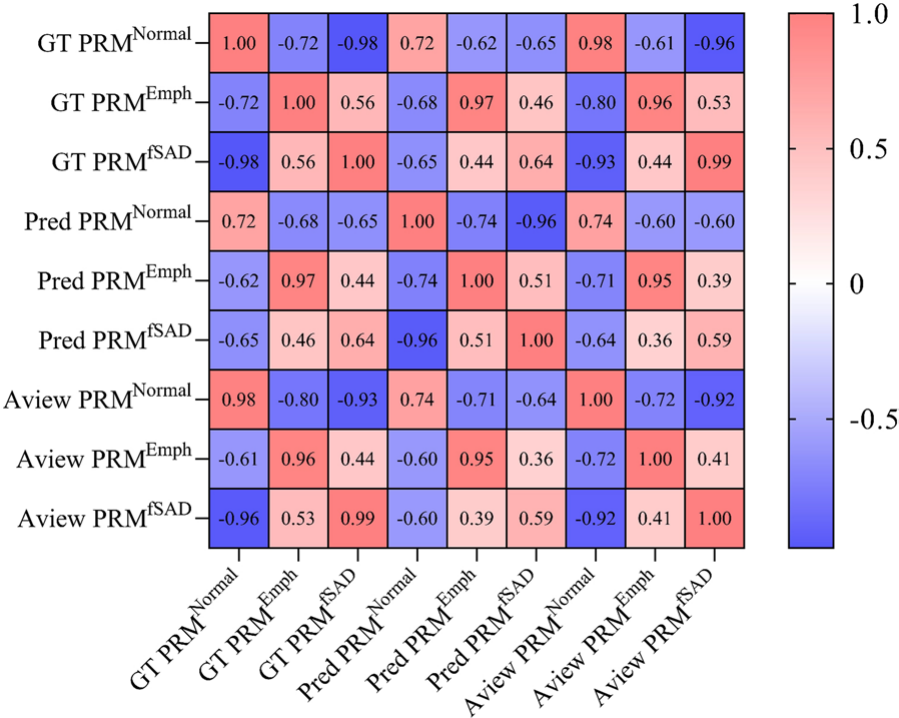
Pearson correlation matrix of quantitative results. PRM: parametric response mapping; GT: ground truth; fSAD: functional small airway disease; PRM^Normal^: the volume percentage of normal area in PRM; PRM^fSAD^: the volume percentage of fSAD in PRM; PRM^Emph^: the volume percentage of emphysema in PRM

In addition to assessing the overall structure of the generated images, Fig. 5 and Additional file 1: Table S3 show the correlation of lesion volume percentages from different PRM sources. The ground truth and the Aview PRM in this study have high consistency in the volume percentage of normal lung tissue, emphysema, and fSAD (|r| values of 0.98, 0.96, and 0.99 respectively, *p* < 0.05), indicating the reliability of the ground truth PRM. The predicted PRM emphysema volume percentage (Pred PRM^Emph^) has high consistency with GT PRM^Emph^ (|r|= 0.97, *p* < 0.05), while the predicted PRM fSAD volume percentage (PRM^fSAD^) shows moderate correlation with GT PRM^fSAD^ (|r|= 0.64, *p* < 0.05). This shows that our model has high similarity in structure and strong correlation in quantitative results compared to the ground truth PRM.

Moreover, both the ground truth and predicted PRM parameters showed significant correlations (*p* < 0.05) with indicators in visual evaluation, such as CLE score, airway morphology, and arterial morphology (Additional file 1: Table S4). This not only reflects the clinical value of the PRM method and aligns with previous pathological studies [11, 28], but also underscores the effectiveness of the predicted PRM.

### Stratification based on PRM^fSAD^

**Fig. 6:**
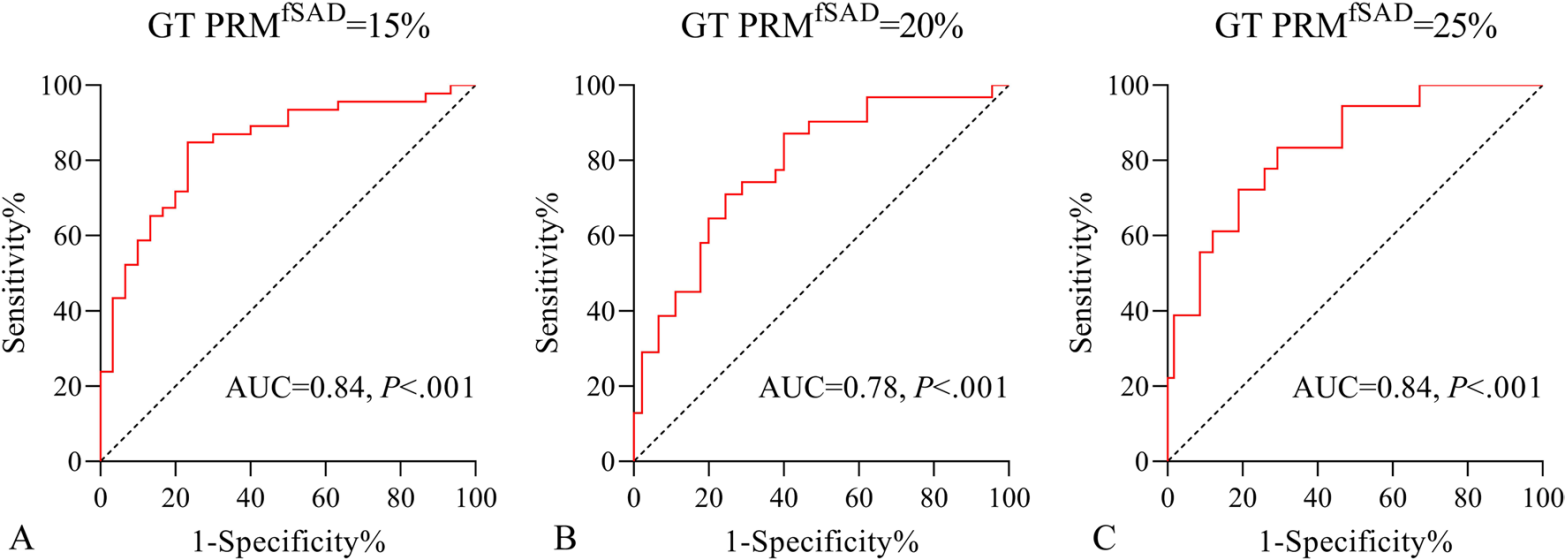
ROC curves of Pred PRM^fSAD^. After using different thresholds (GT PRM^fSAD^ is 15%, 20%, and 25% respectively) for SAD stratification (**A**–**C**), Pred PRM^fSAD^ showed excellent classification performance. AUC: area under the curve; ROC: receiver operating characteristic; GT: ground truth; PRM: parametric response mapping; fSAD: functional small airway disease

As depicted in Fig. 6 and Additional file 1: Table S5, when GT PRM^fSAD^ adopts thresholds of 15%, 20%, and 25% for SAD stratification, the AUC of Pred PRM^fSAD^ is respectively 0.84 (95% CI 0.75–0.93; *p* < 0.001), 0.78 (95% CI 0.68–0.89; *p* < 0.001), and 0.84 (95% CI 0.73–0.94; *p* < 0.001). The cut-off values are respectively 22.8% (sensitivity 0.848, specificity 0.767), 23.2% (sensitivity 0.871, specificity 0.6), and 25.9% (sensitivity 0.833, specificity 0.707), exhibiting robust classification performance.

Using the GT PRM^fSAD^ threshold of 15% stratification, 46 participants were classified as high PRM^fSAD^ and 30 as low PRM^fSAD^ (Tables 1, 3). The incidence of inflammatory SAD was significantly higher in the high PRM^fSAD^ group (54.3%) compared to the low PRM^fSAD^ group (13.3%) (Table 1). The severity of CLE was also more pronounced in the high PRM^fSAD^ group (CLE score: 1.2 ± 1 VS 0.7 ± 1; *p* = 0.045), with CLE comprising 71.7% within the high PRM^fSAD^ group, and CLE excluding trace CLE reaching 36.9%, exceeding the low PRM^fSAD^ group (16.6%) (Table 1). Both tracheal coronal and sagittal diameters were larger in the high PRM^fSAD^ group compared to the low PRM^fSAD^ group (Table 3). Additionally, the proportion of bronchiectasis and paraseptal emphysema was also higher in the high PRM^fSAD^ group. Pred PRM^fSAD^ stratified based on a cut-off value of 22.8%, classified 45 participants as high PRM^fSAD^ and 31 as low PRM^fSAD^ (Table 3, Additional file 1: Table S5), similar to GT PRM^fSAD^ in terms of visual evaluation, with higher incidences of inflammatory SAD, emphysema, bronchiectasis, and wall thickening in the high PRM^fSAD^ group than in the low PRM^fSAD^ group (Table 3).

**Table 3:** Characteristics of test participants stratified by PRM^fSAD^

	GT PRM^fSAD^ > 15%	GT PRM^fSAD^ ≤ 15%	*p*-value	Pred PRM^fSAD^ > 22.8%	Pred PRM^fSAD^ ≤ 22.8%	*p*-value
No. of subjects	46	30	…	45	31	…
Sex, Female, No. (%)	16 (34.8)	13 (43.3)	0.453	14 (31.1)	15 (48.4)	0.128
Age, y	63.8 ± 11.5	58.9 ± 9.3	0.056	62.5 ± 11.7	60.9 ± 9.7	0.55
BMI, kg/m^2^	22.7 ± 2.8	24.7 ± 3.3	0.007	22.4 ± 2.6	25.1 ± 3.2	< 0.001
Chronic cough or phlegm, No. (%)	5 (10.9)	3 (10)	0.904	4 (8.9)	4 (12.9)	0.709
FEV1 (%)	98.1 ± 8	101.3 ± 12.4	0.176	99.3 ± 8.3	99.3 ± 12.2	0.999
FEV1/FVC (%)	88.6 ± 6.6	87.4 ± 8.3	0.483	89.8 ± 5.9	85.7 ± 8.5	0.023
Pred PRM^fSAD^	28.2 ± 6.7	19.2 ± 6.2	< 0.001	…	…	…
Inflammatory SAD, No. (%)	25 (54.3)	4 (13.3)	< 0.001	22 (48.9)	7 (22.6)	0.02
Bronchial wall thickening, No. (%)	2 (4.3)	2 (4.3)	0.645	4 (8.9)	0 (0)	0.141
Bronchial dilation, No. (%)	17 (37)	7 (23.3)	0.212	18 (40)	6 (19.4)	0.057
CLE score	1.2 ± 1	0.7 ± 1	0.045	1.2 ± 1	0.8 ± 1	0.13
Paraseptal emphysema, No. (%)	16 (34.8)	5 (16.7)	0.084	13 (28.9)	8 (25.8)	0.768
Panlobular emphysema, No. (%)	2 (4.3)	1 (3.3)	0.658	2 (4.4)	1 (3.2)	0.789
Tracheal coronal diameter (cm)	1.7 ± 0.2	1.6 ± 0.2	0.022	1.7 ± 0.2	1.6 ± 0.2	0.012
Tracheal sagittal diameter (cm)	1.9 ± 0.3	1.7 ± 0.3	0.031	1.9 ± 0.3	1.7 ± 0.3	0.019
Pulmonary artery diameter (cm)	2.6 ± 0.3	2.6 ± 0.3	0.42	2.5 ± 0.3	2.7 ± 0.3	0.046
Aortic diameter (cm)	3.3 ± 0.4	3.4 ± 0.5	0.932	3.3 ± 0.4	3.4 ± 0.5	0.165
SSIM	0.87 ± 0.04	0.86 ± 0.03	0.248	0.86 ± 0.04	0.87 ± 0.03	0.154

Data are mean ± SD unless indicated otherwise

BMI: body mass index; FEV1: forced expiratory volume in the first second; FVC: forced vital capacity; PRM: parametric response mapping; fSAD: functional small airway disease; PRM^fSAD^: the volume percentage of fSAD in PRM; GT: ground truth; CLE: centrilobular emphysema; SSIM: structural similarity index

## Discussion

In an innovative approach, we utilized a deep learning algorithm to effectively generate PRM from a single inspiratory chest CT scan. The predicted PRM^fSAD^ and PRM^Emph^ demonstrated a fair correlation with the ground truth results. On the other hand, both the ground truth and the predicted PRM^fSAD^ correlate with the visual evaluation of emphysema and airway morphology in HRCT, displaying a similar distribution of lung structure abnormalities when risk stratifying SAD. Using GT PRM^fSAD^ to stratify SAD at thresholds of 15%, 20%, and 25% [15, 25–27], predicted PRM^fSAD^ displayed exceptional classification performance (AUCs of 0.84, 0.78, and 0.84 respectively; *p* < 0.001), which is sufficient for routine SAD clinical screening.

SAD is an early pathological change that occurs before obvious lung structural alterations, spirometry abnormalities, or symptoms appear. Studies have found that respiratory symptoms are common and related to SAD in people with normal spirometry, especially smokers, and that HRCT shows airway changes precede emphysema [29, 30]. We also found that 63.2% of participants with normal spirometry and a history of tobacco smoke exposure had varying degrees of emphysema, and HRCT showed that about one-third had bronchiectasis and inflammatory SAD, indicating that spirometry cannot timely respond to early lung changes. This is because the small airways have a strong reserve capacity, and the injuries need to accumulate to a certain degree before they manifest as symptoms, detectable lung structure or spirometry abnormalities. Once there are factors such as infection, they can easily progress to irreversible obstructive lung disease [5, 31–33], which is also why there’s an urgent need for reliable methods to assess small airways. Besides spirometry, other methods for assessing small airways, such as plethysmography and inert gas washout, are limited by their complexity and difficulty in obtaining equipment, while bronchial provocation tests may cause severe bronchospasm and are also restricted in clinical practice [34, 35].

CT imaging, limited by resolution, cannot directly observe small airways less than 2 mm. However, inflammatory exudation in small airways can be observed on HRCT. Based on PRM^fSAD^, our risk stratification reveals a 50% incidence of inflammatory SAD in the high PRM^fSAD^ group, significantly surpassing the low PRM^fSAD^ group. However, this doesn’t capture the early stages of small airway conditions. When functional impairment of small airways occurs, gas cannot be exhaled normally in the lungs, appearing as abnormally low attenuation areas in the lung parenchyma on expiratory CT, described as air trapping, which can be used to measure the functional status of small airways. Conventional inspiratory CT cannot distinguish between emphysematous air trapping and small airway disease air trapping [36, 37]. Hersh et al. [38] found that indicators such as LAA-856 (the lung voxel percentage with less than − 856 HU on expiratory CT scans) and E/I (the ratio of average lung attenuation on expiratory and inspiratory CT scans) have a weak correlation with emphysema. These measurements based on paired inspiratory and expiratory CT can help better distinguish air trapping due to different pathological bases and can be used as indicators to assess SAD in smokers.

Relying solely on LAA-856 cannot distinguish the source of air trapping components, while E/I cannot provide spatial information about disease distribution. Galbán et al. [11] proposed PRM through attenuation signals on paired inspiratory-expiratory CT scans at the voxel level, dividing the lungs into normal, emphysematous, and fSAD regions. This not only identifies and quantifies different pathological bases of air trapping components but also provides their spatial distribution, which is its unique advantage. PRM^fSAD^ has been proven to have a good correlation with lung function indicators such as FEV1, FEV1/FVC, total lung capacity, and residual volume [11, 25, 39]. Vasilescu et al. [28] proved through lung tissue micro-CT that PRM^fSAD^ is closely related to the loss, narrowing, and obstruction areas of terminal bronchioles.

However, the necessity for PRM to perform both inspiratory and expiratory CT scans doubles the radiation dose and scanning time, and also increases the economic burden, limiting its widespread clinical application. In this study, we combined Generative Adversarial Networks (GANs) and encoder-decoder models (U-Net) for the first time to generate effective PRM without expiratory CT scans. Building on the classification work of Ho et al. [17] and Bodduluri et al. [26], which utilized deep learning for COPD, our method advances this by pinpointing and quantifying distinct gas trapping components within three-dimensional images. Although the dice coefficient of the predicted fSAD and emphysema areas are slightly lower (0.51 and 0.63 respectively), they have similar dice coefficients to the results of Yu et al. [40], who also used the U-Net model. This is mainly because our model generating three-dimensional images of whole lung, which necessitates predicting more voxels in terms of location, shape, or density. This presents a more challenging task. In the future, image section learning segmentation can also be considered [41], which may improve dice, but will also lose three-dimensional spatial information. It is worth noting that compared with real PRM, the generated PRM has more fSAD in non-dependent lung areas and less in dependent lung areas (Fig. 4). We speculate that this may be related to differences in exhalation levels during CT scanning among participants. During exhalation, the density of dependent lung areas increases more significantly than non-dependent lung areas [42, 43], and insufficient exhalation can lead to incorrect density signal distribution between the two areas. Although expiratory scans are not required during generation, their supervision signals are included in model training, affecting the quality of generated images. Despite respiratory training and exclusion of unqualified images, subtle differences in exhalation levels between participants cannot be avoided. Considering that this cannot be completely eliminated in real application scenarios, it is acceptable for the model's performance to decline when predicting fSAD. Overall, our model has good feasibility and innovation, fills a gap in this field, and provides a promising direction for small airway assessment and screening.

There were some limitations to our research. Firstly, due to the small sample size and single-center nature of the study, we were unable to obtain external validation data to assess the robustness and applicability of the model. Secondly, considering the target population of our inclusion criteria, our model is mainly applicable to populations with relatively normal spirometry; we plan to include participants with varying degrees of lung impairment in future studies to improve the generalizability of the model. In addition, the lack of respiratory gating in CT scanning may lead to inaccuracies in image generation.

Regarding model technology, we found that the predicted PRM results by the U-Net are smooth, while the generated PRM with the expiratory HU threshold is easily influenced by image noise. Both of these methods are hardly consistent with the real PRM results. Although we proposed to integrate the generated PRM from the U-Net and threshold results to improve the results to be more realistic and accurate, the distribution of the generated results slightly differs from the real image. Moreover, due to the instability of GAN model training and the limitations of data distribution, the expiratory CT generated from inspiratory CT may exhibit mode collapse, resulting in false positives in the PRM prediction area obtained from a small number of healthy test samples. In the future, we can try to use data augmentation or self-supervised pre-training to improve the generalization performance. Due to the low resolution and noise problem, we found that GAN model is easily affected by the CT quality, which makes it more difficult to train a stable and easily converging model. We chose to use cropped patches from the inspiratory and registered expiratory CT scans to reduce the training difficulty. As the diffusion model [44, 45] proposed recently, we can use the diffusion model to replace the GAN model to improve the robustness of the generation model. Furthermore, as the diffusion model can synthesize data from noisy inputs, we will further implement a diffusion model to obtain more available training data, construct larger datasets with synthetic high-resolution CT. Finally, our proposed method uses a multi-stage network, which means extra training time and cumulative errors, and we will further implement end-to-end training and inference in the future to achieve better performance.

## Conclusion

By utilizing only inspiratory chest CT images, we have proposed a deep learning method capable of generating PRM. This method successfully achieves both qualitative and quantitative imaging diagnosis of fSAD and emphysema at the voxel level in populations of smokers or those exposed to secondhand smoke with normal spirometry, while also excelling in stratified screening for SAD. Furthermore, the use of only inspiratory CT reduces both radiation dose and economic cost for patients, thus enabling large-scale screening for SAD and providing a promising approach for early COPD screening.

### Supplementary Information

**Additional file 1. Visual Assessment on HRCT.  Construction of PRM Generative Model. Table S1.** The AUC of Pred PRM^fSAD^ at Different Thresholds of GT PRM^fSAD^ (n = 76). **Table S2.** Characteristics of Enrolled Participants. **Table S3.** Pearson Correlation Coefficients of PRM Metrics (n = 76). **Table S4.** Pearson Correlation Coefficient of HRCT Visual Evaluation and PRM Metrics (n = 76). **Table S5.** The AUC of Pred PRM^fSAD^(n = 76).

## Data Availability

The datasets used and/or analysed during the current study are available from the corresponding author on reasonable request.

## Abbreviations

ADE: Advanced destructive emphysema
AUC: Area under the curve
BMI: Body mass index
CLE: Centrilobular emphysema
COPD: Chronic obstructive pulmonary disease
FEV1: Forced expiratory volume in the first second
fSAD: Functional small airway disease
FVC: Forced vital capacity
GAN: Generative adversarial networks
GT: Ground truth
HRCT: High resolution computed tomography
PRM: Parametric response mapping
PRM^Emph^: The volume percentage of emphysema in PRM
PRM^fSAD^: The volume percentage of fSAD in PRM
PRM^Normal^: The volume percentage of normal area in PRM
RMSE: Root mean squared error
ROC: Receiver operating characteristic
SAD: Small airway disease
SSIM: Structural similarity index
