# Supplementary material for: Deep learning parametric response mapping from inspiratory chest CT scans: a new approach for small airway disease screening
Source: Respir Res. 2023 Nov 28;24:299. doi: 10.1186/s12931-023-02611-2 (PMC10683250; doi:10.1186/s12931-023-02611-2)
Supplement: Supplementary file 1 — Additional file 1. Visual Assessment on HRCT. Construction of PRM Generative Model. Table S1. The AUC of Pred PRMfSAD at Different Thresholds of GT PRMfSAD (n = 76). Table S2. Characteristics of Enrolled Participants. Table S3. Pearson Correlation Coefficients of PRM Metrics (n = 76). Table S4. Pearson Correlation Coefficient of HRCT Visual Evaluation and PRM Metrics (n = 76). Table S5. The AUC of Pred PRMfSAD(n = 76). [file 12931_2023_2611_MOESM1_ESM.docx]

**Additional file**

# *Visual Assessment on HRCT*

We adhered to the Fleischner Society statement[1] for the visual assessment and scoring of emphysema, bronchial wall thickness, bronchiectasis, and small airway disease in participants’ lung HRCT scans. For detailed descriptions of lesion characteristics, please refer to the Fleischner Society statement[1]. Bronchial wall thickening was defined as a bronchial wall thickness greater than 50% of the diameter of adjacent pulmonary arteries outside the hilar region, while bronchiectasis was measured by an airway-artery diameter ratio >1. Additionally, we conducted a semi-quantitative assessment of the extent of bronchiectasis and small airway disease lesions using three degrees: single, multiple, and extensive lung segments. CT images were independently evaluated by two radiologists (a senior radiologist with more than 10 years of experience and a junior radiologist with more than 5 years of experience) who were blinded to the participants’ clinical information. Any discrepancies in their evaluations were discussed and resolved through consensus.

**Centrilobular Emphysema (CLE):** Emphysema assessment is performed at window position -700 to -900HU and window width 750 to 1000HU. The scoring is as follows:

No emphysema, score as 0;

Trace CLE, score as 1;

Mild CLE, score as 2;

Moderate CLE, score as 3;

Confluent CLE, score as 4;

Advanced Destructive Emphysema (ADE), score as 5.

**Panlobular Emphysema:** Visually assessed as present or absent.

**Paraseptal Emphysema**

No paraseptal emphysema, score as 0;

Mild paraseptal emphysema, score as 1;

Substantial paraseptal emphysema, score as 2.

**Bronchial wall thickening:** Visually assessed as present or absent.

**Bronchiectasis**

No bronchiectasis, score as 0;

Bronchiectasis in one lung segment, score as 1;

Bronchiectasis in multiple lung segments, score as 2;

Bronchiectasis in extensive lung segments, score as 3.

**Inflammatory Small Airway Disease (SAD):** Inflammation within and around small airways can result in their clear visibility on CT, manifesting as ill-defined ground-glass centrilobular nodules[2, 3].

No inflammatory SAD, score as 0;

Inflammatory SAD involving one lung segment, score as 1;

Inflammatory SAD involving multiple lung segments, score as 2;

Inflammatory SAD involving extensive lung segments, score as 3.

**Tracheal coronal diameter:** The coronal diameter of the trachea at the level of the upper edge of the aortic arch was measured on inspiratory chest CT.

**Tracheal sagittal diameter:** The sagittal diameter of the trachea at the level of the upper edge of the aortic arch was measured on inspiratory chest CT.

**Pulmonary artery diameter and aortic diameter:** Pulmonary and aortic diameters were measured on transverse CT at the level of the pulmonary artery bifurcation, where the left and right pulmonary arteries form a right angle.

:

# *Construction of PRM Generative Model*

**Reconstructing expiratory CT scan**

Generative adversarial networks (GANs) are recently widely used to construct the generative model. However, one deep network may not generate a CT scan with sufficient clear texture features. To decrease the difficulty of model fitting the mapping function, we divide the whole generator into two networks.

The first GAN generates the intermediate registered expiratory CT scan from inspiratory domain. The inspiratory CT scan and temporary registered expiratory CT scan from the first GAN are fed into another conditional GAN to refine expiratory CT scan prediction. Similar to the structure of other GANs, our model consists of two adversarial models: a generative model G that generate the registered expiratory CT scan and a discriminative model D that distinguishes the real and generated expiratory CT scan.

The training process of G and D can be summarized as the process of game balance. G is expected to generate the expiratory CT scan that confuse D. Therefore, we adopt the adversarial loss function to optimize our model:

$$\min_{G_{1}} \max_{D_{1}} L\left( D_{1}, G_{1} \right)=\mathbb{E}_{z\sim P_{data}(z)}\left[ \log D_{1}\left( z \right) \right]+\mathbb{E}_{x\sim P_{data}(x)}[log(1-D_{1}(G_{1}(x)))]$$

$$\min_{G_{2}} \max_{D_{2}} L\left( D_{2}, G_{2} \right)=\mathbb{E}_{z\sim P_{data}(z)}\left[ \log D_{2}\left( z \right) \right]+\mathbb{E}_{x\sim P_{data}(x)}[log(1-D_{2}(G_{2}(x|G_{1}(x))))]$$

Where $G_{1}$ and $D_{1}$ are the generator and discriminator in the first network, $G_{2}$ is the conditional generator, $D_{2}$ is the discriminator of $G_{2}$, $P_{data}(x)$ and $P_{data}(z)$ are the distribution of inspiratory and expiratory CT scans.

To close the gap between the generated and the real expiratory CT scan, we use $L_{1}$ norm:

$$L_{reg}(G) =\mathbb{E}[||G(x)-z||_{1}]$$

The final loss function is defined as:

$$L\left( G, D \right)=L\left( D, G \right)+\alpha* L_{reg}(G)$$

**PRM Generator**

In light of the difference between generated and real expiratory CT scan, it’s hard to get the similar predicted PRM compared to the label PRM with the HU threshold of -856. We found the local HU biases of generated expiratory CT scan influence the results. To decrease the influence of noise in generating images, we generate PRMs from two perspectives to ensure the stability of the results. We construct a segmentation network like U-Net model to directly predict the label of each voxel in CT scan. In addition, the features in the segmentation network are inputted into MLP to get the threshold for generated expiratory CT scan. Lastly, the PRM predicted by segmentation network and the PRM generated by the predicted expiratory threshold are integrated to get the final PRM prediction.

In this paper, we choose the cross-entropy loss[4] to train the segmentation network as:

$$L_{c}= -\Sigma_{j=1}^{c}\Sigma_{v\in V}y_{jv}\log(\hat{y}_{jv})$$

Where C is the number of labels, y is the ground truth PRM, $\hat{y}$ is the predicted PRM, V represents the voxel set in the CT scans.

The dice loss is introduced in our model to alleviate the class imbalance problem. The dice loss can be represented as:

$$L_{dice}^{s}=\Sigma_{c=0}^{C}1-\frac{\left| Y_{c}\bigcap\hat{Y}_{c} \right|}{\left| Y_{c} \right|+\left| \hat{Y}_{c} \right|}$$

Where $\left| Y_{c}\bigcap\hat{Y}_{c} \right|$ is the number of pixels in the overlapping area of label $c$, $\left| Y_{c} \right|$ and $\left| \hat{Y}_{c} \right|$ are the number of label $c$ pixels in the target and predicted PRM.

To deal with the voxel value distribution deviation of the generated expiratory phase, we add a MLP layer from the bottom feature of segmented network to regress a trainable threshold. With the threshold $\eta$ we calculate the probability of lesion as:

$$P_{lesion}=Sigmoid(100*(\eta-G_{2}(x)))$$

Where $\lambda$ is the trained threshold, $G_{2}(x)$ is the generated expiratory CT scans. Lastly, we compute the dice loss of predicted lesion area and ground truth lesion area.:

$$L_{dice}^{t}=1-\frac{\left| P_{lesion}\bigcap\hat{P}_{lesion} \right|}{\left| P_{lesion} \right|+\left| {\hat{P}_{lesion}}_{c} \right|}$$

For the predicted threshold and segmentation map are both from the PRM generation network, the final loss can be represented as:

$$L_{seg}=L_{c}+\beta*L_{dice}^{s}+\gamma*L_{dice}^{t}$$

**Implementation details**

To avoid out-of-memory (OOM) errors, we crop the CT scans to several 128×128×128 patches. The inspiratory, expiratory and PRM CT scans are all crop to patches of the same size using the same cropping strategy. At least half the height, weight or depth of patches are overlapped by neighbor patches. A voxel in PRM could be decided by different patches. For each patch, voxels at the center of the patch have higher confidence. Thus, we multiply probability output of patches by the gaussian kernel to emphasize the weight of the center voxels and weaken the weight of the edge voxels. Lastly, all weighted patches are concatenated together to reconstruct the predicted PRM of the original size.

In the training stage, we first train the expiratory generator until the network convergence. To alleviate the over-fitting problem, we employ the data argumentation like adding Gaussian noise and Gaussian blurring. We adopted the stochastic gradient descent algorithm with a momentum of 0.99 and an initial learning rate of 0.01 as the optimizer. The parameter α, β and γ are all set to 0.1.

**References:**

1. Lynch DA, Austin JH, Hogg JC, Grenier PA, Kauczor HU, Bankier AA, Barr RG, Colby TV, Galvin JR, Gevenois PA *et al*: **CT-Definable Subtypes of Chronic Obstructive Pulmonary Disease: A Statement of the Fleischner Society.** *RADIOLOGY* 2015, **277**(1):192-205.

2. Okada F, Ando Y, Yoshitake S, Ono A, Tanoue S, Matsumoto S, Wakisaka M, Maeda T, Mori H: **Clinical/pathologic correlations in 553 patients with primary centrilobular findings on high-resolution CT scan of the thorax.** *CHEST* 2007, **132**(6):1939-1948.

3. Gruden JF, Webb WR: **CT findings in a proved case of respiratory bronchiolitis.** *AJR. American journal of roentgenology* 1993, **161**(1):44-46.

4. Xu H, Yang M, Deng L, Qian Y, Wang C: **Neutral Cross-Entropy Loss Based Unsupervised Domain Adaptation for Semantic Segmentation.** *IEEE transactions on image processing : a publication of the IEEE Signal Processing Society* 2021, **30**:4516-4525.

| **Table S1** The AUC of Pred PRM^fSAD^ at Different Thresholds of GT PRM^fSAD^ (n = 76) | | | | | | | | | | | | | | | | | | | | | |
| --- | --- | --- | --- | --- | --- | --- | --- | --- | --- | --- | --- | --- | --- | --- | --- | --- | --- | --- | --- | --- | --- |
| GT PRM^fSAD^ (%) | 10 | 11 | 12 | 13 | 14 | 15 | 16 | 17 | 18 | 19 | 20 | 21 | 22 | 23 | 24 | 25 | 26 | 27 | 28 | 29 | 30 |
| AUC | 0.80 | 0.80 | 0.80 | 0.83 | 0.85 | 0.84 | 0.84 | 0.83 | 0.81 | 0.78 | 0.78 | 0.77 | 0.76 | 0.82 | 0.83 | 0.84 | 0.85 | 0.81 | 0.81 | 0.80 | 0.79 |
| *PRM*, parametric response mapping; *GT*, ground truth; *fSAD*, functional small airway disease; *PRM^fSAD^*, the volume percentage of fSAD in PRM; *AUC*, area under the curve. | | | | | | | | | | | | | | | | | | | | | |

| **Table S2** Characteristics of Enrolled Participants | | | | |
| --- | --- | --- | --- | --- |
|  | Total | Training set | Tuning set | Test set |
| No. of subjects | 537 | 381 | 80 | 76 |
| Sex, Female, No. (%) | 263 (49) | 194 (50.9) | 40 (50) | 29 (38.2) |
| Age, y | 62.2 ± 9.3 | 62.3 ± 8.9 | 62.5 ± 9.5 | 61.8 ± 10.9 |
| BMI, kg/m^2^ | 23.7 ± 3.3 | 23.7 ± 3.4 | 24.1 ± 3.2 | 23.5 ± 3.1 |
| Chronic cough or phlegm, No. (%) | 64 (11.9) | 48 (12.6) | 7 (8.8) | 9 (11.4) |
| FEV1 (%) | 102.7 ± 13.6 | 104.7 ± 14.8 | 102.3 ± 15.5 | 99.3 ± 10 |
| FEV1/FVC (%) | 88.6 ± 7.1 | 88.9 ± 6.9 | 87.9 ± 7.3 | 88.1 ± 7.3 |
| Data are mean ± SD unless indicated otherwise.  *BMI*, body mass index; *FEV1*, forced expiratory volume in the first second; *FVC*, forced vital capacity. | | | | |

| **Table S3** Pearson Correlation Coefficients of PRM Metrics (n = 76) | | | | | | | | | |
| --- | --- | --- | --- | --- | --- | --- | --- | --- | --- |
| Quantitative results | Aview PRM^fSAD^ | Aview PRM^Emph^ | Aview PRM^Normal^ | GT PRM^fSAD^ | GT PRM^Emph^ | GT PRM^Normal^ | Pred PRM^fSAD^ | Pred PRM^Emph^ | Pred PRM^Normal^ |
| Aview PRM^fSAD^ | 1.00* | 0.41* | -0.92* | 0.99* | 0.53* | -0.96* | 0.59* | 0.39* | -0.60* |
| Aview PRM^Emph^ | 0.41* | 1.00* | -0.72* | 0.44* | 0.96* | -0.61* | 0.36* | 0.95* | -0.60* |
| Aview PRM^Normal^ | -0.92* | -0.72* | 1.00* | -0.93* | -0.80* | 0.98* | -0.64* | -0.71* | 0.74* |
| GT PRM^fSAD^ | 0.99* | 0.44* | -0.93* | 1.00* | 0.56* | -0.98* | 0.64* | 0.44* | -0.65* |
| GT PRM^Emph^ | 0.53* | 0.96* | -0.80* | 0.56* | 1.00* | -0.72* | 0.46* | 0.97* | -0.68* |
| GT PRM^Normal^ | -0.96* | -0.61* | 0.98* | -0.98* | -0.72* | 1.00* | -0.65* | -0.62* | 0.72* |
| Pred PRM^fSAD^ | 0.59* | 0.36* | -0.64* | 0.64* | 0.46* | -0.65* | 1.00* | 0.51* | -0.96* |
| Pred PRM^Emph^ | 0.39* | 0.95* | -0.71* | 0.44* | 0.97* | -0.62* | 0.51* | 1.00* | -0.74* |
| Pred PRM^Normal^ | -0.60* | -0.60* | 0.74* | -0.65* | -0.68* | 0.72* | -0.96* | -0.74* | 1.00* |
| * indicates Pearson correlation coefficient *p* < 0.05.  *PRM*, parametric response mapping; GT, ground truth; *fSAD*, functional small airway disease; *PRM^Normal^*, the volume percentage of normal area in PRM; *PRM^fSAD^*, the volume percentage of fSAD in PRM; *PRM^Emph^*, the volume percentage of emphysema in PRM. | | | | | | | | | |

| **Table S4** Pearson Correlation Coefficient of HRCT Visual Evaluation and PRM Metrics (n = 76) | | | | | | | | | | | | |
| --- | --- | --- | --- | --- | --- | --- | --- | --- | --- | --- | --- | --- |
|  | GT PRM^Emph^ | Pred PRM^Emph^ | GT PRM^fSAD^ | Pred PRM^fSAD^ | Inflammatory SAD | Bronchial wall thickening | CLE score | Bronchial dilation | Pulmonary artery diameter | Aortic diameter | Tracheal coronal diameter | Tracheal sagittal diameter |
| GT PRM^Emph^ | … | … | … | … | 0.11 | 0.05 | 0.42* | 0.46* | -0.09 | -0.07 | -0.07 | 0.38* |
| Pred PRM^Emph^ | … | … | … | … | 0.05 | 0.02 | 0.42* | 0.45* | -0.13 | -0.13 | -0.07 | 0.38* |
| GT PRM^fSAD^ | … | … | … | … | 0.45* | 0.06 | 0.24* | 0.30* | -0.10 | -0.02 | 0.22 | 0.39* |
| Pred PRM^fSAD^ | … | … | … | … | 0.20 | 0.12 | 0.30* | 0.27* | -0.30* | -0.27* | 0.18 | 0.34* |
| Inflammatory SAD | 0.11 | 0.05 | 0.45* | 0.20 | 1.00 | 0.18 | 0.13 | -0.05 | -0.12 | -0.03 | 0.14 | 0.14 |
| Bronchial wall thickening | 0.05 | 0.02 | 0.06 | 0.12 | 0.18 | 1.00 | 0.06 | 0.05 | -0.06 | -0.02 | -0.18 | 0.06 |
| CLE score | 0.42* | 0.42* | 0.24* | 0.30* | 0.13 | 0.06 | 1.00 | 0.10 | 0.13 | 0.13 | -0.06 | 0.45* |
| Bronchial dilation | 0.46* | 0.45* | 0.30* | 0.27* | -0.05 | 0.05 | 0.10 | 1.00 | 0.32* | 0.12 | 0.11 | 0.27* |
| Pulmonary artery diameter | -0.09 | -0.13 | -0.10 | -0.30* | -0.12 | -0.06 | 0.13 | 0.32* | 1.00 | 0.61* | -0.10 | 0.13 |
| Aortic diameter | -0.07 | -0.13 | -0.02 | -0.27* | -0.03 | -0.02 | 0.13 | 0.12 | 0.61* | 1.00 | -0.02 | 0.33* |
| Tracheal coronal diameter | -0.07 | -0.07 | 0.22 | 0.18 | 0.14 | -0.18 | -0.06 | 0.11 | -0.10 | -0.02 | 1.00 | 0.06 |
| Tracheal sagittal diameter | 0.38* | 0.38* | 0.39* | 0.34* | 0.14 | 0.06 | 0.45* | 0.27* | 0.13 | 0.33* | 0.06 | 1.00 |
| * indicates Pearson correlation coefficient *p* < 0.05.  *PRM*, parametric response mapping; GT, ground truth; *PRM^Emph^*, the volume percentage of emphysema in PRM; *fSAD*, functional small airway disease; *PRM^Normal^*, the volume percentage of normal area in PRM; *PRM^fSAD^*, the volume percentage of fSAD in PRM; *CLE*, centrilobular emphysema. | | | | | | | | | | | | |

| **Table S5** The AUC of Pred PRM^fSAD^ (n = 76) | | | | | |
| --- | --- | --- | --- | --- | --- |
| GT PRM^fSAD^ (%) | AUC (95%CI) | *p-*value | Cut off for Pred PRM^fSAD^ (%) | Sensitivity | Specificity |
| 15 | 0.84 (0.75-0.93) | <0.001 | 22.8 | 0.848 | 0.767 |
| 20 | 0.78 (0.68-0.89) | <0.001 | 23.2 | 0.871 | 0.6 |
| 25 | 0.84 (0.73-0.94) | <0.001 | 25.9 | 0.833 | 0.707 |
| *PRM*, parametric response mapping; *GT*, ground truth; *fSAD*, functional small airway disease; *PRM^fSAD^*, the volume percentage of fSAD in PRM; *AUC*, area under the curve. | | | | | |
